# Effects of different pressure midfoot wraps on balance and proprioception in amateur basketball athletes

**DOI:** 10.3389/fbioe.2025.1560522

**Published:** 2025-05-23

**Authors:** Chengliang Wu, Shuai Zhang, Tao Wu, Sheng-Wei Jia, Zhaowei Chu, Fan Yang

**Affiliations:** ^1^ Department of Sports Rehabilitation, School of Sports Medicine, Wuhan Sports University, Wuhan, China; ^2^ Biomechanics Laboratory, Li Ning Sports Science Research Center (LN-SSRC), Beijing, China

**Keywords:** midfoot, balance, proprioception, compression, electromyography

## Abstract

**Introduction:**

Ankle sprains are prevalent in basketball. This study sought to determine how midfoot wraps affect postural stability and ankle proprioception.

**Methods:**

Twenty-two amateur basketball athletes performed three single-leg balance tests (static, head-elevated static, and unstable foam pad) under four wrap conditions (no wrap, low, medium, and high pressure), and balance measures were taken using a force platform. Standing time, center of pressure dynamics, surface electromyographic of the supporting leg musculature were recorded. Ankle proprioception joint position matching error was assessed by a digital inclinometer.

**Results and discussion:**

Results indicated that during balance tests on foam padding, participants demonstrated significantly longer standing time when wearing low-pressure midfoot wraps, compared to high-pressure wraps (*F* (3,63) = 4.32, *p* = 0.008, *η* ^2^ = 0.17). Wearing high-pressure wraps reduced anterior-posterior dynamic stability index variability (*F* (3,63) = 3.89, *p* = 0.044, *η* ^2^ = 0.16), suggesting enhanced sagittal-plane control. Intriguingly, high-pressure conditions evidenced convergent activation trends between medial and lateral gastrocnemius (GM/GL ratio shift from 1.3 to 1.0), albeit without statistical significance (*p* > 0.05). No significant difference was detected in joint position sense in ankle dorsiflexion, plantarflexion, eversion and inversion between different wrap conditions (*p* > 0.05). These findings suggest that low-pressure midfoot wraps may improve balance through enhanced cutaneous feedback, while high-pressure wraps enhance anterior-posterior dynamic stability, providing biomechanically informed strategies for ankle injury prevention in basketball.

## Introduction

Basketball, characterized by high-intensity movements such as rapid acceleration, jumping, and abrupt directional transitions, imposes places significant biomechanical demands on ankle stability and postural control (Ferioli et al., 2023; Wen et al., 2018). Ankle sprains account for 15%–25% of all basketball-related injuries (McKay et al., 2001; Fong et al., 2007), with impaired proprioception during destabilized landings identified as a primary risk factor (Huynh et al., 2021). Notably, ankle sprains are often classified separately from other foot injuries (e.g., midfoot ligament strains, metatarsal fractures) based on their unique anatomical location and injury mechanisms (Gribble et al., 2016). Despite the high prevalence of non-ankle foot injuries—evidenced by basketball’s highest severe foot injury rate (IR = 10.71 per 100,000 athlete-exposures) in male collegiate athletes (Chan et al., 2021)—existing interventions (e.g., ankle braces) predominantly target large joints, neglecting the midfoot’s critical role in sensorimotor integration during dynamic movements. The anatomical connection between proprioception and joint stability is primarily achieved through the integration of receptors and structural continuity (Ghai et al., 2018), with the midfoot being an important component of the foot.

Compression garments, which are engineered to apply controlled mechanical pressure on specific anatomical regions, have demonstrated potential to enhance athletic performance via augmented proprioception (Martorelli et al., 2015) and dynamic stability (Woo et al., 2018). Empirical evidence highlights their benefits, including enhanced muscle activation, reduced soft-tissue vibration (Deng et al., 2021), and refined proprioceptive feedback (Sperlich et al., 2013; Zamporri and Aguinaldo, 2018). Soft-tissue vibration is defined as mechanical oscillations in muscles and connective tissues during dynamic movements, which can disrupt proprioceptive feedback and neuromuscular control (Deng et al., 2021). Compression garments are widely utilized in various sports to reduce fatigue and enhance athletic performance. For example, compression socks have been shown to improve running performance and maintain higher vertical jump heights in runners wearing medium and low compression socks during a 10-km run (Ali et al., 2011; Kemmler et al., 2009). Unlike ankle-spanning compression garments or rigid ankle-foot orthoses that restrict joint mobility (Chan et al., 2021; Esposito et al., 2015), midfoot wraps specifically target the longitudinal arch’s sensory motor integration without compromising athletic performance—a critical distinction for basketball’s dynamic demands (Ma et al., 2023). Collectively, these findings underscore the potential of compression garments to enhance athletic performance by improving proprioception, balance, and muscle function (Macrae et al., 2011). Their biomechanical effects—particularly those modulating proprioception and dynamic stability—are paramount for sports requiring rapid directional changes, such as basketball (Macrae et al., 2011; Zamporri and Aguinaldo, 2018). However, research in basketball has predominantly focused on fatigue recovery, while midfoot-specific compression remains underexplored.

Proprioceptive feedback, mediated by cutaneous mechanoreceptors and muscle spindles, plays a critical role in maintaining postural equilibrium during dynamic motor tasks (Han et al., 2015). Currently, the majority of research on compression garments in basketball has predominantly focused on fatigue recovery, with limited attention paid to proprioception and balance function. Proprioception exhibits a strong correlation with athletic performance, as evidenced by higher proprioception scores and superior ankle angle discrimination abilities in athletes compared to the general population (Han et al., 2014; Han et al., 2015). Although exercise therapy and taping can enhance balance, midfoot wraps offer a non-invasive and task-specific solution that directly targets cutaneous mechanoreceptors without compromising athletic performance (Deschamps et al., 2018).

The midfoot exerts a critical influence in both biomechanical stability and proprioceptive feedback during basketball-specific movements. Biomechanically, the midfoot’s longitudinal arch plays a pivotal role in postural control by distributing ground reaction forces and maintaining foot alignment during dynamic tasks such as landing (Olsen et al., 2019). Proprioceptively, the midfoot’s dense network of cutaneous mechanoreceptors facilitates real-time feedback on foot orientation and load distribution, thereby enabling precise adjustments to maintain balance (Kavounoudias et al., 1998). Impaired proprioceptive acuity has been directly associated to ankle instability (Hou et al., 2024), underscoring the need to explore how midfoot-specific compression can enhance these mechanisms. However, current interventions (e.g., ankle braces) primarily focus on large joints, neglecting the midfoot’s dual roles in biomechanical stability and sensorimotor integration (Deschamps et al., 2018). This oversight highlights two key research gaps: 1. How midfoot-specific compression influences postural stability during dynamic tasks. 2. Whether targeted compression enhances proprioceptive acuity to reduce injury risks.

This study seeks to examine the effects of varying midfoot wrap pressures on balance and proprioception in amateur basketball athletes. We formulated the following hypotheses: 1. Low-pressure midfoot wraps will improve static balance on unstable surfaces by enhancing cutaneous feedback. 2. High-pressure wraps will enhance dynamic stability in the anterior-posterior plane through proprioceptive recalibration. 3. Midfoot compression will not directly influence ankle joint proprioception due to region-specific sensory dissociation.

## Methods

### Participants

The required sample size was estimated using G*power software (Version 3.1.9.7) for a one-way repeated measures ANOVA (within-subjects factor: four pressure conditions), based on an effect size (*η* ^2^ = 0.30) derived from a pilot study (*n* = 10) examining midfoot wrap effects on standing time. Input parameters included a statistical power of 0.85, *α* = 0.05, and a correlation among repeated measures of 0.5 (Faul et al., 2007). This analysis yielded a total sample size of 22 participants. *Post-hoc* power analysis confirmed adequate statistical power (1-β > 0.80) for all significant findings (Cohen, 1988; Faul et al., 2007). Twenty-two participants (16 males and 6 females) were recruited via convenience sampling from local amateur basketball leagues, with an average age of 23.8 ± 1.9 years, height of 173 ± 8 cm, and weight of 68.8 ± 15.3 kg. Participants were screened for neuromuscular disorders, cardiovascular disease, and recent (<6 months) lower-limb injuries (Hong et al., 2022). None reported a history of ankle sprains or regular use of medications/caffeine/alcohol affecting balance (Huxham et al., 2001). Each participant was informed about the experimental procedure and provided a signed informed consent form. Ethical approval was granted by the Ethics Committee of the Wuhan Sports University, adhering to the Declaration of Helsinki.

### Midfoot wrap pressure selection

**FIGURE 1:**
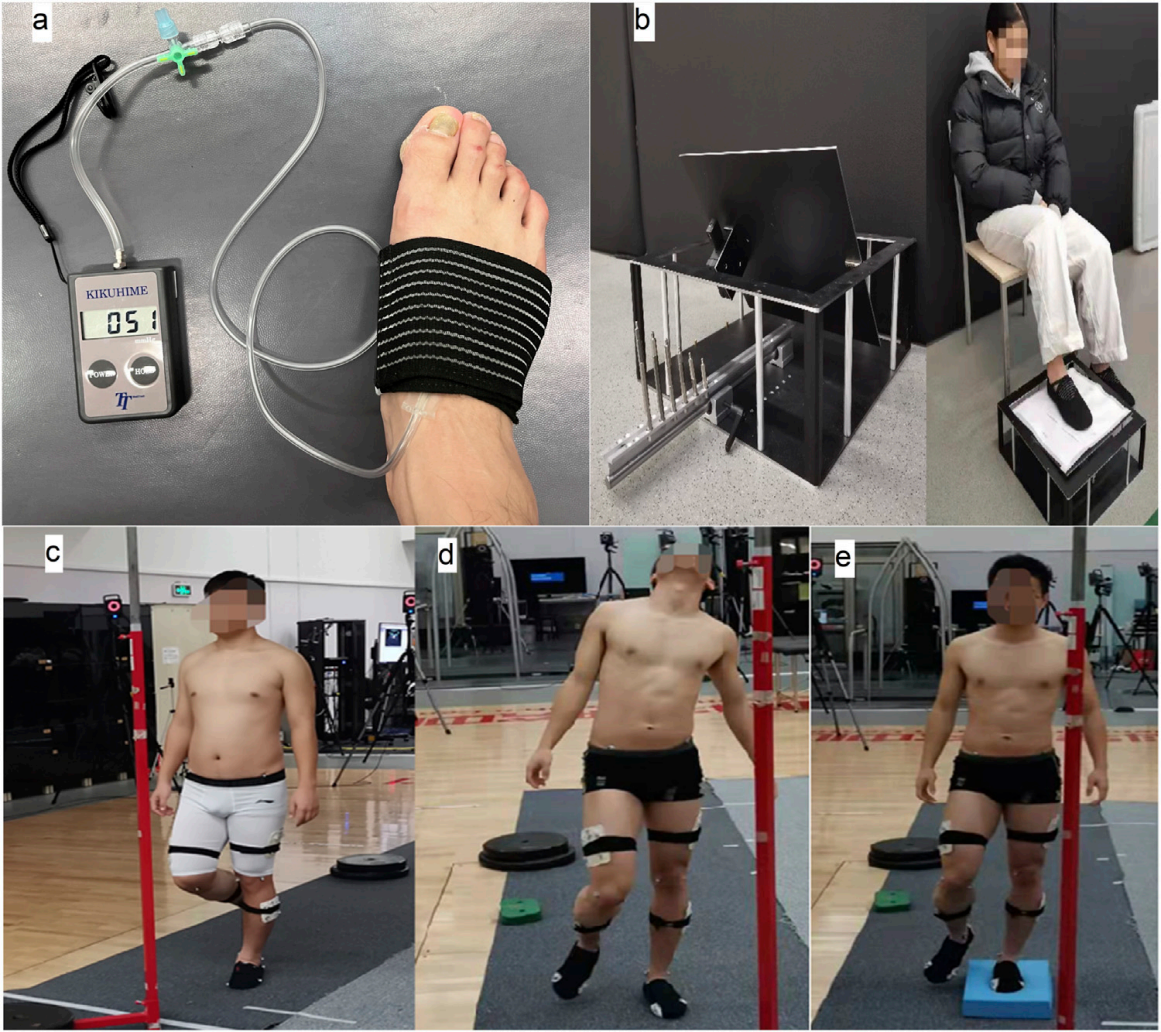
Diagram of midfoot wraps **(a)**, Ankle proprioception test **(b)**, Single-leg standing with eyes closed **(c)**, standing with head up and eyes closed **(d)**, and standing on a foam pad with eyes closed **(e)**.

The midfoot anatomically delineated as the region spanning from the talonavicular joint proximally to the tarsometatarsal joints distally, comprising the navicular, cuboid, and three cuneiform bones (DiDomenico and Thomas, 2014). Pressure intensities were systematically established using a dual-phase protocol that integrated subjective comfort assessments and objective pressure quantification. The Danish Kikuhime pressure monitor (HPM–KH-01; accuracy: ±8 mmHg) was weekly validated against certified balibration standards using a digital dynamometer, with the transducer positioned at the navicular tuberosity (Figure 1a). The elastic bandage (McDavid Self-Adherent Wrap) was wrapped circumferentially around the midfoot with three overlapping layers, guaranteeing uniform pressure distribution across the midfoot region. The “medium-pressure” condition (denoted as F) was operationally defined 46**–**56 mmHg (mean ± SD: 51.1 ± 4.2 mmHg), derived from participant-reported comfort levels ≥7/10 on a visual analog scale (VAS) **(** Warenczak et al., 2014). Low- (0.5F: 23–29 mmHg, mean ± SD: 25.6 ± 3.0 mmHg) and high-pressure (1.5F: 69–84 mmHg, mean ± SD: 76.7 ± 5.5 mmHg) conditions were selected based on mechanoreceptor activation thresholds reported in prior studies (Zhang et al., 2015), thereby achieving differential afferent stimulation while preserving functional joint mobility.

### Balance test

Three single-leg balance protocols (static, head-elevated static, and unstable surface) were systematically selected to represent a gradient of difficulty, mimicking progressive challenges to postural control demands characteristic of basketball movements maneuvers (Wikstrom et al., 2005):

(1) Static balance: Eyes closed on a firm surface.
(2) Static balance (head elevated): Eyes closed with head elevated 30° (to disrupt vestibular input).
(3) Unstable surface: Eyes closed on a 10-cm-thick foam pad (Airex Balance Pad, 2020). The unstable foam pad configuration replicates surface irregularity patterns frequently encountered in outdoor basketball environments or rehabilitation scenarios.

Two practice trials per condition were administered to ensure participant familiarization with experimental procedures. Participants stood on a 3D force platform (AMTI BP12001200, 1,000 Hz) with arms relaxed (Figures 1c–e). Each condition was repeated twice under four wrap conditions (no-wrap, low-, medium-, high-pressure), randomized to minimize order effects. Standing time, center of pressure (COP) metrics, and surface EMG signals (Noraxon, 2000 Hz) from the tibialis anterior (TA), medial gastrocnemius (GM), lateral gastrocnemius (GL), and soleus (SO) were recorded. Electrode placement adhered to SENIAM standards, maintaining a fixed inter-electrode spacing of 20 mm (Hermens et al., 2000). A 5-min rest period was enforced between conditions to minimize fatigue. Trials were repeated if participants lost balance (e.g., touched the ground with the raised foot).

### The ankle proprioception test

Active joint position sense was quantified through a modified seated weight-bearing paradigm adapted from established methodology (Han et al., 2016). With visual input occluded, participants executed active ankle mobilization to four predetermined reference angles (20° inversion, 25° eversion, 10° dorsiflexion, 15° plantarflexion) presented in randomized order, held for 5 s to enhance proprioceptive memory (Zhang et al., 2011). This seated, vision-occluded protocol was designed to isolate somatosensory afferent signals by eliminating confounding visual and vestibular inputs (Han et al., 2016), thereby prioritizing internal validity for mechanistic insights into midfoot compression effects. After returning to neutral, participants performed active joint position reproduction by replicating each angle three times (Figure 1b). Angular discrepancies were measured using a digital inclinometer (Goniometer Pro, ±1° accuracy).

### Data processing

Participants naturally stood on the force platform. Upon hearing the “start” cue, they lifted their non-dominant foot, while the researcher started the stopwatch. Timing stopped when the supporting foot moved or the lifted foot touched the ground. Two trials were conducted per participant, with the trial demonstrating optimal postural maintenance selected for subsequent analysis, recorded in seconds and rounded to one decimal place. COP metrics: Maximum displacement and average velocity were calculated in anterior-posterior (AP) and medial-lateral (ML) directions using AMTI Net Force software. CoP velocity served as the principal balance outcome measure, reflecting its established sensitivity to postural adjustments in anterior-posterior and medial-lateral directions (Wikstrom et al., 2005). Maximum displacement amplitude was computed by subtracting the minimum displacement from the maximum displacement in the anterior-posterior or medial-lateral direction of the COP. Additionally, the average COP velocity was determined as the mean displacement across frames, calculated as (the displacement of the current frame minus the displacement of the previous frame) divided by the inverse of the sampling frequency.

The Dynamic Postural Stability Index (DPSI), derived from force platform data during single-leg stance, quantifies dynamic balance by integrating fluctuations in ground reaction forces across three planes. The DPSI was calculated as described by Wikstrom et al. (2005), incorporating three components: anterior-posterior stability index (APSI), medial-lateral stability index (MLSI), and vertical directions stability index (VSI). These three parameters are computed using the mean squared deviation to quantify fluctuations in the ground reaction force data set around zero, calculated as follows (Equations 1–4):

$$\text{MLSI}=\sqrt{\frac{\sum {\left(\frac{Fx}{body\;weight}\right)}^{2}}{number\;of\;data\;points}}$$ (1)

$$\text{APSI}=\sqrt{\frac{\sum {\left(\frac{Fy}{body\;weight}\right)}^{2}}{number\;of\;data\;points}}$$ (2)

$$\text{VSI}=\sqrt{\frac{\sum {\left(\frac{body\;weight-Fz}{body\;weight}\right)}^{2}}{number\;of\;data\;points}}$$ (3)

$$\text{DPSI}=\sqrt{\frac{\sum {\left(\frac{Fx}{body\;weight}\right)}^{2}+\sum {\left(\frac{Fy}{body\;weight}\right)}^{2}+\sum {\left(\frac{body\;weight-Fz}{body\;weight}\right)}^{2}}{number\;of\;data\;points}}$$ (4)

The triaxial force components (Fx: medial-lateral, Fy: anterior-posterior, Fz: vertical) corresponding to ground reaction forces were acquired via the force platform. Data acquisition windows were standardized to 3-s epochs. A lower DPSI score indicates better dynamic balance, reflecting smaller fluctuations in ground reaction forces, while a higher DPSI score suggests poorer dynamic balance due to larger fluctuations (Wikstrom et al., 2005). The force platform operated at a sampling frequency of 1,000 Hz, generating 3,000 discrete observations per 3-s trial.

EMG processing: Raw surface EMG signal were subjected to full-wave rectification followed by band-pass filtered (10–400 Hz) in accordance with established protocols (van Dieen et al., 2009). Integrated electromyography (IEMG) quantifies both motor unit recruitment and firing rate characteristics (Winter, 2009), and IEMG is calculated as follows (Equation 5):

$$IEMG=\int_{t}^{t+T}\left|EMG\left(t\right)\right|\cdot dt$$ (5)

Here, *t* denotes the start time and *t* + *T* represents the end time of the EMG signal. Muscle contribution refers to the ratio of the activation level of a specific muscle to the IEMG sum of the muscles measured in completing this movement during a single leg balance. It indicates the importance of the muscle in completing the action (Winter, 2009).

Ankle proprioception was quantified as the absolute angular deviation between the reproduced angle and the target reference angle (e.g., 20° inversion, 25° eversion). Three replication trials per target angle were conducted, with proprioceptive performance indexed by the mean absolute error across trials (Han et al., 2016).

### Statistical analysis

A one-way repeated measures ANOVA design was implemented to assess the main effects of midfoot compression gradients on single-leg balance and proprioception, with the Bonferroni method used for *post hoc* analysis. Eta-squared (η^2^) effect size magnitudes were interpreted using conventional thresholds: 0.14 (large), 0.06 (medium), and 0.01 (small) (Cohen, 1988). Normality was confirmed via Shapiro-Wilk tests (*p* > 0.05 for all variables). All results were expressed as mean ± standard deviation, with a significance threshold set at *α* = 0.05.

## Results

### Balance kinematics

**FIGURE 2:**
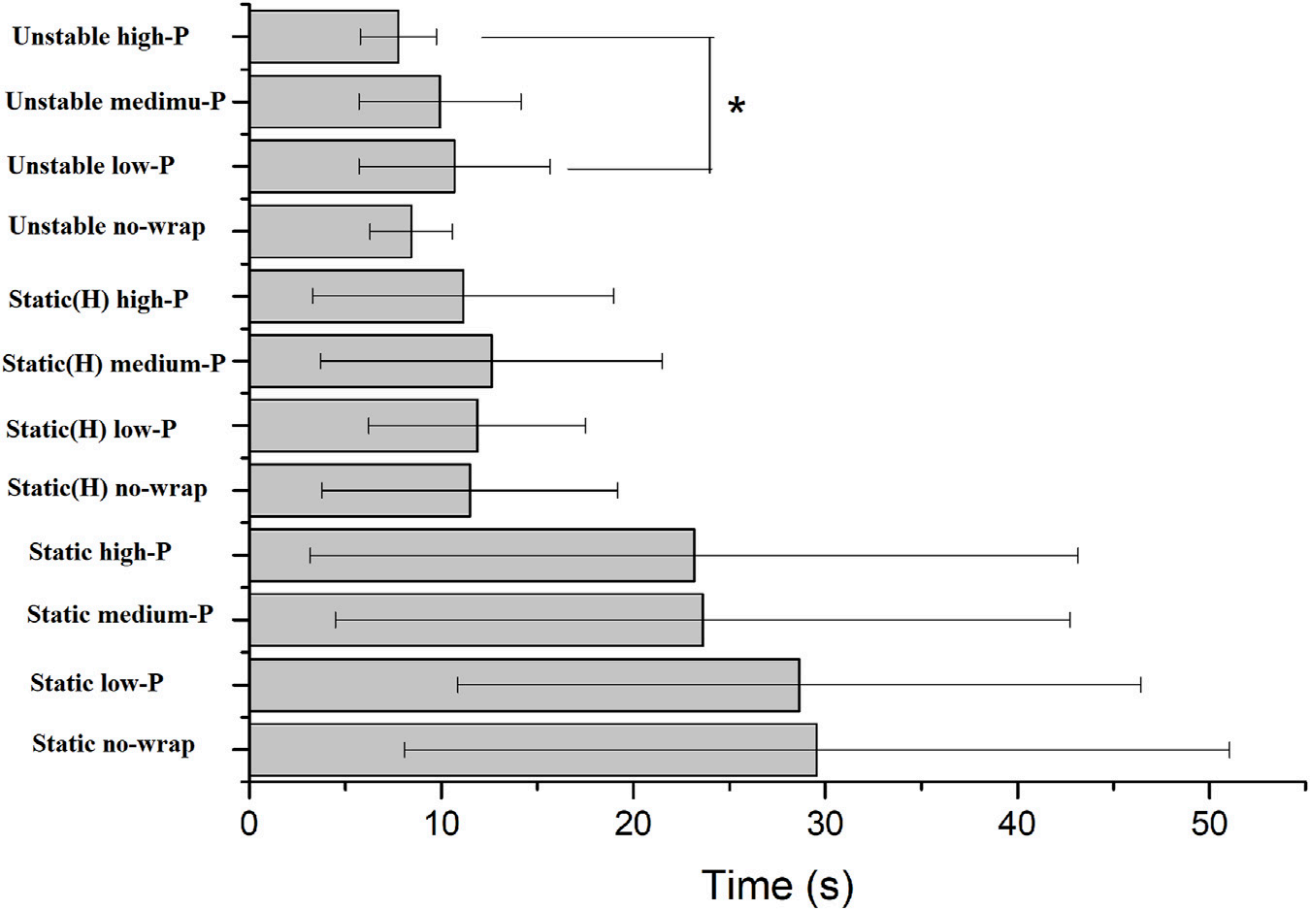
Standing time of single-leg balance test. Bars indicate group means; error bars = SD. *Indicates significant, *p* < 0.05 (low-P vs. high-P). Static, Static (H), and Unstable represent the three types of single-leg balance tests (Static balance, Static balance [head elevated], Unstable surface). No-wrap, low-P, medium-P, and high-P refer to the four midfoot wrapping conditions (no-wrap, low-pressure, medium-pressure, high-pressure).

The single-leg balance test included three conditions: eyes closed, head-up with eyes closed, and foam-padded stance with eyes closed. A one-way repeated measures ANOVA revealed no statistically significant main effects of midfoot wrap pressure on standing time for the first two conditions (eyes closed: *F* (3,63) = 1.12, *p* = 0.347, *η* ^2^ = 0.05; head-up: *F* (3,63) = 0.93, *p* = 0.431, *η* ^2^ = 0.04). However, under the foam-padded condition, a significant of pressure was observed (*F* (3,63) = 4.32, *p* = 0.008, *η* ^2^ = 0.17), with *post hoc* tests indicating longer standing times in low-pressure wraps conditions compared to high-pressure conditions (*p* = 0.003, 95% CI [1.2, 4.8 s]; Figure 2).

**FIGURE 3:**
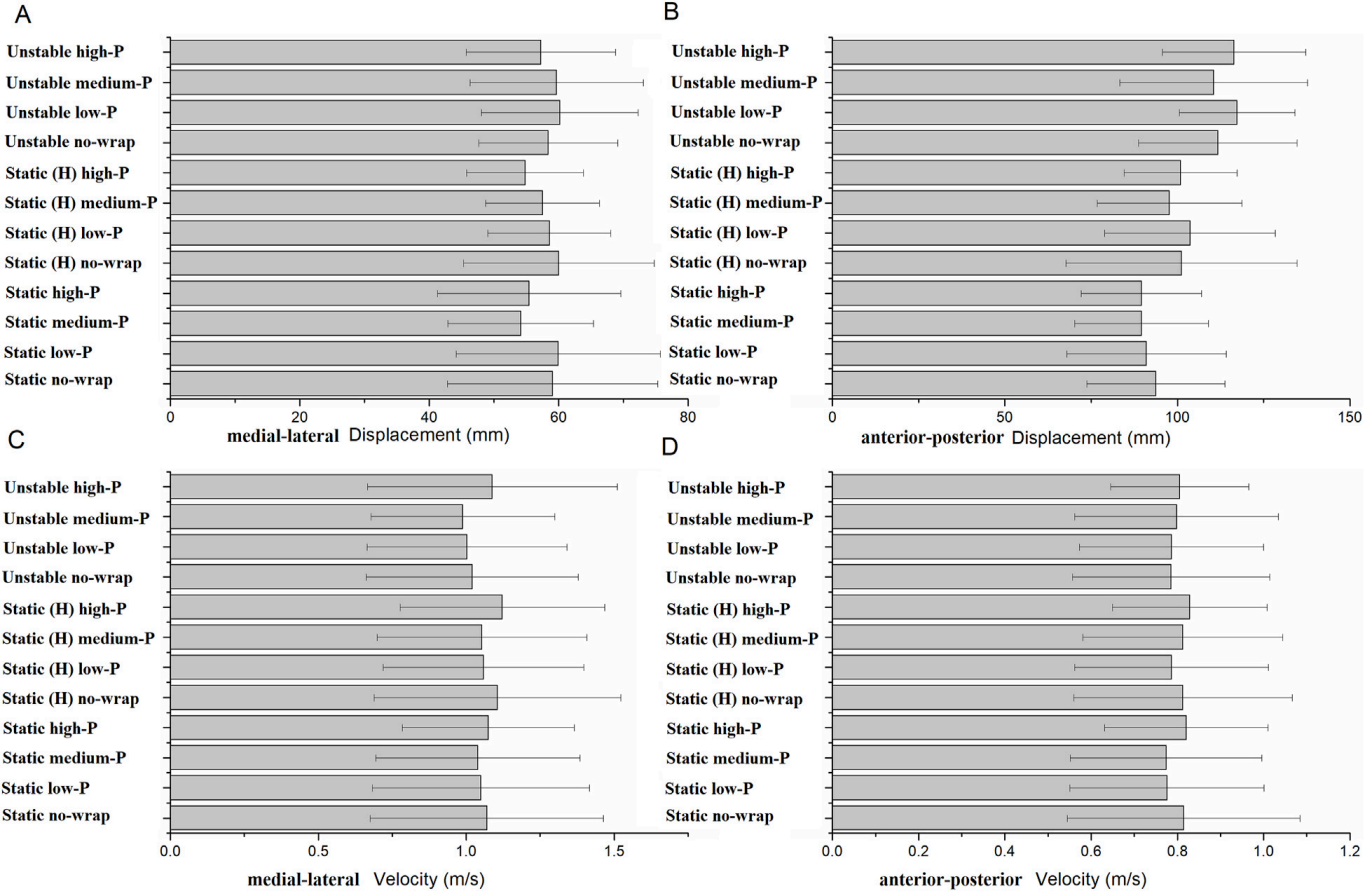
Maximum displacement and average velocity of the center of pressure during the single-leg balance test. Bars indicate group means; error bars = SD. **(A–D)** represent the maximum displacement and average velocity of the center of pressure in the medial-lateral and anterior-posterior directions, respectively. Static, Static (H), and Unstable represent the three types of single-leg balance tests (Static balance, Static balance [head elevated], Unstable surface). No-wrap, low-P, medium-P, and high-P refer to the four midfoot wrapping conditions (no-wrap, low-pressure, medium-pressure, high-pressure).

No significant difference was detected in the maximum displacement and average velocity of the COP across midfoot pressure conditions of the midfoot in the following three single-leg balance tests (*p* > 0.05, *η* ^2^ < 0.06) (Figure 3).

### Dynamic balance

A significant pressure effect was identified for the anterior-posterior stability index (APSI) during single-leg closed-eye stance (*F* (3,63) = 3.98, *p* = 0.044, *η* ^2^ = 0.16), with high-pressure wraps showing reduced APSI values relative to no-wrap (*p* = 0.012) and low-pressure (*p* = 0.025) conditions (Table 1). No significant differences were found for DPSI, MLSI, or VSI (all *p* > 0.05, η^2^ < 0.06).

**TABLE 1:** Dynamic postural stability index and directional components (mean ± SD) with statistical values.

Action	Variable	No wrap	Low pressure	Medium pressure	High pressure	*F* (3,63)	*p*-value	*η* ^*2*^
Static balance	DPSI	0.043 ± 0.042	0.034 ± 0.012	0.033 ± 0.010	0.035 ± 0.012	1.12	0.369	0.05
	APSI	0.014 ± 0.007^a^	0.015 ± 0.009^b^	0.011 ± 0.004	0.011 ± 0.004	**3.98**	**0.044**	**0.16**
	MLSI	0.016 ± 0.005	0.014 ± 0.003	0.014 ± 0.006	0.014 ± 0.005	0.73	0.534	0.03
	VSI	0.035 ± 0.043	0.026 ± 0.011	0.025 ± 0.011	0.028 ± 0.012	1.07	0.367	0.05
Static balance (head elevated)	DPSI	0.046 ± 0.042	0.040 ± 0.011	0.038 ± 0.013	0.041 ± 0.011	0.75	0.574	0.03
	APSI	0.015 ± 0.007	0.016 ± 0.006	0.013 ± 0.006	0.014 ± 0.005	1.12	0.345	0.05
	MLSI	0.017 ± 0.007	0.018 ± 0.005	0.018 ± 0.008	0.018 ± 0.005	0.18	0.849	0.01
	VSI	0.037 ± 0.043	0.031 ± 0.012	0.030 ± 0.012	0.032 ± 0.012	0.81	0.513	0.04
Unstable surface	DPSI	0.054 ± 0.039	0.045 ± 0.016	0.047 ± 0.017	0.048 ± 0.015	0.97	0.43	0.04
	APSI	0.014 ± 0.007	0.014 ± 0.005	0.013 ± 0.005	0.012 ± 0.003	0.88	0.453	0.04
	MLSI	0.022 ± 0.009	0.020 ± 0.007	0.023 ± 0.009	0.022 ± 0.009	1.14	0.361	0.05
	VSI	0.045 ± 0.040	0.037 ± 0.015	0.038 ± 0.016	0.040 ± 0.014	0.85	0.459	0.04

Static balance, Static balance (head elevated), and Unstable surface represent the three single-leg balance test conditions in this study. *F* (3,63) refers to the degrees of freedom (with 3 degrees of freedom for the treatment effect and 63 for the error). Significance notation: Bold indicates *p* < 0.05. Superscript letters (a, b) denote *post hoc* test results: a: No Wrap vs. High Pressure. b: Low Pressure vs. High Pressure.

### Electromyographic (EMG) activity

**FIGURE 4:**
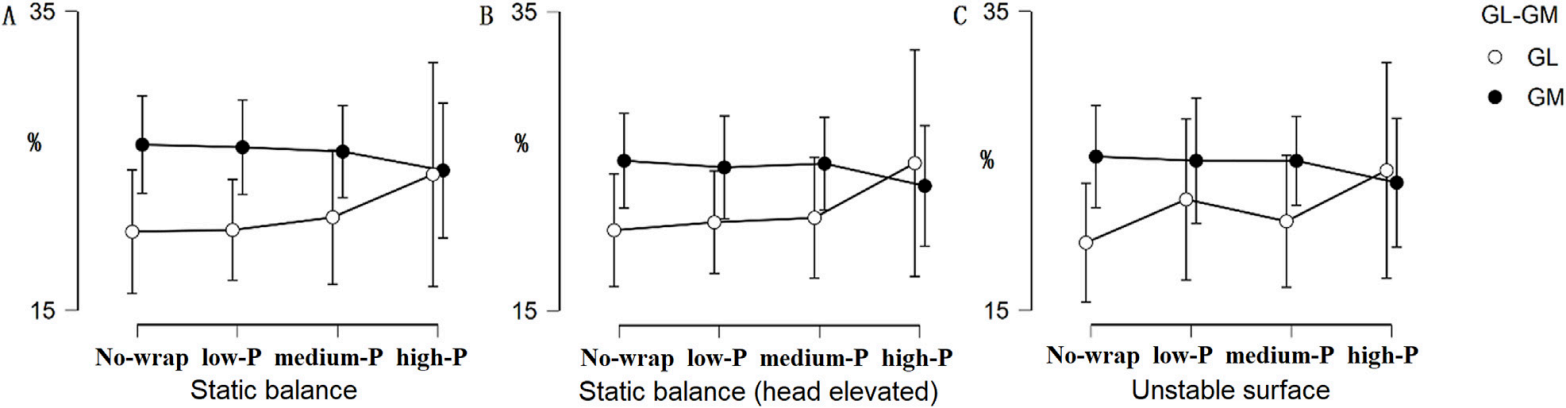
Changes in the contribution of GL and GM during the single-leg balance test. Solid and hollow dots both indicate group means, error bars = SD. No-wrap, low-P, medium-P, and high-P refer to the four midfoot wrapping conditions (no-wrap, low-pressure, medium-pressure, high-pressure). **(A)** Static balance; **(B)** Static balance (head elevated); **(C)** Unstable surface.

Muscle contribution ratios showed no significant pressure effects (Table 2, all *p* > 0.05, η^2^ < 0.08). Notably, high-pressure conditions demonstrated converging activation trends between medial and lateral gastrocnemius (GM/GL ratio shift from 1.3 to 1.0), though these differences did not reach statistical significance (*p* > 0.05, η^2^ < 0.06) (Figure 4).

**TABLE 2:** Muscle contribution levels (%, mean ± SD).

Action	Muscle	No wrap	Low pressure	Medium pressure	High pressure	*F* (3,63)	*p*-value	*η* ^*2*^
Static balance	TA	33.9 ± 21.4	33.2 ± 21.1	33.9 ± 23.3	33.4 ± 21.9	0.03	0.931	0.001
	GL	20.3 ± 12.9	20.4 ± 12.1	21.2 ± 13.7	24.1 ± 19.5	0.93	0.437	0.04
	GM	26.1 ± 12.7	25.9 ± 13.1	25.6 ± 13.2	24.4 ± 14.0	0.89	0.426	0.04
	SO	19.8 ± 13.0	20.6 ± 13.2	19.3 ± 13.3	18.2 ± 12.3	1.32	0.276	0.06
Static balance (head elevated)	TA	34.9 ± 20.5	33.9 ± 20.4	34.3 ± 21.1	34.1 ± 22.1	0.17	0.886	0.01
	GL	20.4 ± 11.8	20.9 ± 12.0	21.2 ± 12.7	24.9 ± 19.2	1.14	0.338	0.05
	GM	25.0 ± 13.4	24.6 ± 13.5	24.8 ± 12.8	23.4 ± 14.2	0.95	0.423	0.04
	SO	19.7 ± 12.6	20.6 ± 13.2	19.6 ± 12.3	17.6 ± 12.1	1.83	0.141	0.08
Unstable surface	TA	34.5 ± 19.0	33.2 ± 19.7	33.9 ± 21.0	33.1 ± 20.8	0.45	0.694	0.02
	GL	19.5 ± 11.6	22.4 ± 15.6	21.0 ± 12.4	24.4 ± 19.2	1.07	0.369	0.05
	GM	25.3 ± 13.1	25.0 ± 14.3	25.0 ± 13.5	23.5 ± 13.8	0.84	0.47	0.04
	SO	20.7 ± 13.3	19.4 ± 13.5	20.1 ± 12.9	19.0 ± 13.0	0.93	0.439	0.04

Static balance, Static balance (head elevated), and Unstable surface represent the three single-leg balance test conditions in this study. All *F*-values and η^2^ are based on one-way repeated measures ANOVA (degrees of freedom *F* (3,63)). The η^2^ effect sizes are categorized as small effects (η^2^ < 0.06) or approaching medium effects (0.06 ≤ η^2^ < 0.10).

### Ankle proprioception

**FIGURE 5:**
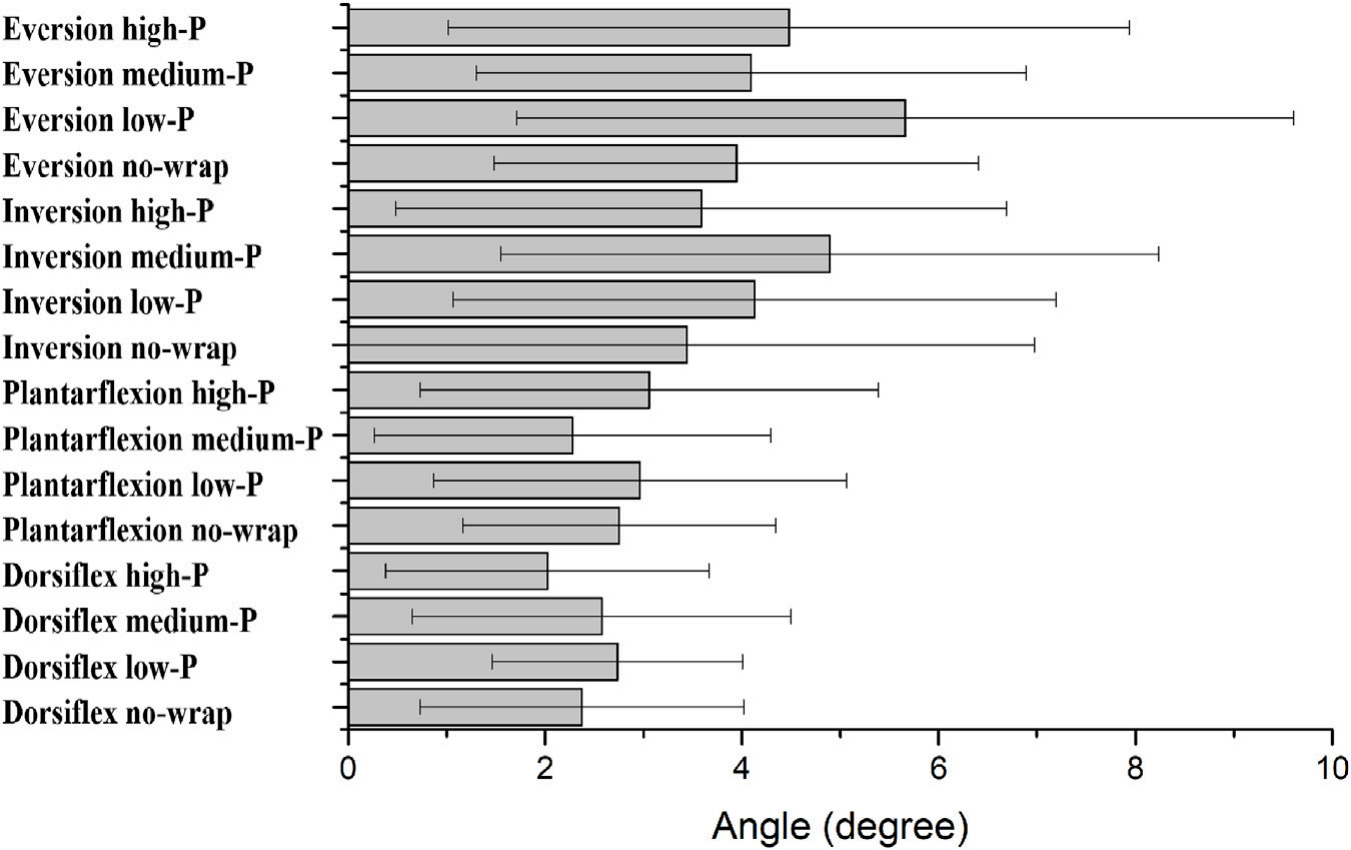
Differences in joint position senses for ankle dorsiflexion, plantarflexion, inversion, and eversion. Bars indicate group means; error bars = SD. No-wrap, low-P, medium-P, and high-P refer to the four midfoot wrapping conditions (no-wrap, low-pressure, medium-pressure, high-pressure).

No significant difference was detected in joint position sense in ankle dorsiflexion, plantarflexion, eversion and inversion between different wrap conditions (all *p* > 0.05, η^2^ < 0.06) (Figure 5).

## Discussion

This study investigated the influence of varying pressure midfoot wraps on balance and proprioception in amateur basketball athletes. The findings reveal that low-pressure compression (0.5F) significantly enhanced unstable-surface balance, while high-pressure (1.5F) selectively improved anterior-posterior dynamic stability by reducing APSI variability. Lower APSI values indicate diminished anterior-posterior ground reaction forces fluctuations, which correlate with improved dynamic stability during single-leg stance (Wikstrom et al., 2005). Despite these improvements, no significant effects were observed on ankle proprioception.

Balance is fundamental for maintaining postural stability during functional activities including static standing, locomotion, and basketball-specific maneuvers (e.g., cutting motions and jumps). Midfoot compression at differing pressure levels did not significantly influence balance duration (*p* > 0.05), except for low-pressure wraps. These significantly prolonged single-leg stance time during eyes-closed trials on an *unstable foam pad* (*η* ^2^ = 0.17, *p* = 0.008; Figure 2). Previous research indicates that compression garments provide negligible balance enhancement for healthy young adults *on* stable surfaces (Cavanaugh et al., 2016; Hijmans et al., 2009; Jaakkola et al., 2017)*,* which aligns with our null findings for no-wrap and medium-pressure conditions. The lack of improvement under medium pressure—despite its subjective comfort (VAS ≥7/10)—may stem from insufficient mechanical stimulation of cutaneous mechanoreceptors to enhance proprioceptive feedback, whereas high-pressure wraps may impose biomechanical constraints on midfoot joint mobility, thereby counteracting potential benefits (Ghai et al., 2018; Zhang et al., 2015)*.* Conversely, low-pressure wraps likely balanced skin stretch and joint motion freedom, optimizing sensory input from the midfoot’s longitudinal arch during unstable stance (Woo et al., 2018). This specificity echoes Woo et al. (2018), who observed compression-enhanced stability only under perturbed conditions. Therefore, low-pressure midfoot wraps may benefit athletes during rehabilitation on uneven terrain or when adapting to novel surfaces, though basketball-specific applications require caution given standardized court hardness.

The human body maintains dynamic stability during single-leg balance, rather than remaining completely stationary. During balance tests, no significant differences were found in the maximum displacement and average velocity of the COP with different midfoot wrapping pressures. Similarly, wearing tights did not affect the average velocity of the COP in young adults during balance tests, primarily because they generally have good balance control (Hijmans et al., 2009). While single-leg stance is traditionally a static measure, the DPSI quantifies dynamic balance by analyzing fluctuations in ground reaction forces across three planes (anterior-posterior, medial-lateral, and vertical) during the stance. The DPSI quantifies dynamic balance, demonstrating highly sensitive to the test results (Wikstrom et al., 2005). Notably, a significant pressure effect was observed exclusively in APSI during single-leg stance (*F* (3,63) = 3.98, *p* = 0.044, *η* ^2^ = 0.16;Table 1), with high-pressure wraps showing reduced APSI variability compared to no-wrap and low-pressure conditions. Lower APSI values indicate reduced anterior-posterior fluctuations in ground reaction forces, reflecting more controlled postural sway and enhanced dynamic stability in the sagittal plane (Wikstrom et al., 2005). This directional specificity may correspond to the sport-specific biomechanical demands of basketball: rapid accelerations and decelerations predominantly challenge anterior-posterior stability, requiring coordinated activation of the gastrocnemius-soleus complex (Stojanović et al., 2018). The selective improvement in anterior-posterior stability reflects basketball’s reliance on sagittal-plane movements (e.g., accelerations and jumps), where midfoot cutaneous feedback optimizes proprioceptive acuity. Conversely, medial-lateral adjustments during stance are limited by compression-induced restrictions in midfoot inversion/eversion mobility (Zhang et al., 2015).

Surface EMG captures neuromuscular activation patterns in lower extremity musculature during balance tasks. No statistically significant differences emerged in muscular recruitment patterns across experimental conditions (all *p* > 0.05, η^2^ < 0.08; Table 2), suggesting that variations in midfoot wrap pressure had a limited impact on overall neuromuscular recruitment patterns. This aligns with Miyamoto et al. (2011), who reported unchanged EMG amplitudes in triceps surae under graduated compression socks, and Fu et al. (2012), who hypothesized that compression-induced muscle performance improvements may operate through peripheral mechanisms (e.g., reduced soft-tissue vibration) rather than central neuromuscular adaptations. Although high-pressure conditions showed a numerical shift in the medial-to-lateral gastrocnemius activation ratio (GM/GL ratio shift from 1.3 to 1.0), this trend did not reach statistical significance. The lack of pressure effects on EMG metrics implies that balance improvements (e.g., APSI reduction with high-pressure wraps) may stem from enhanced cutaneous feedback rather than altered muscle activation strategies (Woo et al., 2018). These observations should be interpreted cautiously due to the study’s limited statistical power to detect subtle neuromuscular changes.

Compression garments can promote proprioception acuity by modulating afferent feedback during motor tasks (Engel and Sperlich, 2016). Chang et al. (2022) found that wearing compression socks positively affected ankle proprioceptive control after running 21 km. However, under seated non-fatigued testing conditions, midfoot compression interventions showed no statistically detectable effects on ankle joint position sense accuracy (all *p* > 0.05; Figure 5). This divergence may be attributed to three mechanistic considerations: (1) The localized midfoot compression in our study primarily stimulated cutaneous mechanoreceptors around the navicular tuberosity (Figure 1), which have weaker neural connectivity to ankle joint capsules compared to ankle-spanning compression garments (Debolt et al., 2024). (2) Proprioceptive enhancements in Chang et al. (2022) emerged post-fatigue, suggesting compression may counteract fatigue-induced sensory degradation—a mechanism absent in our rested participants. (3) The inherent superior ankle proprioception in young athletes (Hijmans et al., 2009), potentially masking subtle effects of midfoot compression. These findings imply that midfoot wraps may be more effective for populations with compromised proprioception (e.g., chronic ankle instability) or during fatigued states. Nevertheless, the lack of ankle proprioceptive improvements aligns with Zhang et al. (2015), who reported no metatarsal wrapping effects on ankle joint position sense, reinforcing the regional specificity of compression benefits.

In summary, this study demonstrated pressure-dependent effects of midfoot wraps on balance but not ankle proprioception in amateur basketball athletes: Low-pressure wraps prolonged single-leg stance duration during eyes-closed trials on unstable surfaces, likely by enhancing cutaneous feedback without restricting midfoot mobility. High-pressure wraps attenuated anterior-posterior stability index variability, suggesting improved dynamic stability through optimized gastrocnemius activation patterns. No proprioceptive improvements were observed, possibly due to localized midfoot stimulation and non-fatigued testing conditions. These findings support task-specific recommendations: Low-pressure wraps are recommended for rehabilitation protocols involving uneven surfaces to optimize balance recovery. Conversely, high-pressure wraps could be considered for high-intensity training or competitions on hard courts to enhance dynamic stability.

### Limitations and future directions

This study has limitations. First, the sample size (*n* = 22) with gender disparity (72.7% males) constrains population generalizability, particularly regarding female athletes and clinical subgroups. Second, the lack of basketball-specific functional tests (e.g., cutting/jumping) and direct sensory data weakens mechanistic interpretation of balance improvements linked to cutaneous feedback. Third, while high-pressure wraps improved APSI, their functional relevance remains unclear without performance metrics. Future studies should focus on expanding cohorts with gender-balanced and clinical subgroups, integrating on-court tasks (e.g., agility drills), and measuring sensory responses to validate feedback mechanisms.

## Conclusion

This study demonstrated that low-pressure midfoot wraps prolonged single-leg stance duration on unstable surfaces, possibly mediated by enhanced cutaneous feedback, while high-pressure wraps enhanced anterior-posterior dynamic stability, as evidenced by reduced APSI variability. No significant changes in ankle proprioception were observed. For ankle sprain prevention, high-pressure wraps may help stabilize linear movements on hard courts, though the effects on lateral agility remain unverified. Low-pressure wraps could be beneficial for balance rehabilitation on uneven terrain. Additionally, this study offers valuable insights for designing midfoot wraps in basketball footwear.

## Data Availability

The original contributions presented in the study are included in the article/supplementary material, further inquiries can be directed to the corresponding author.
